# Study of the Effect of Manganese Ion Addition Points on the Separation of Scheelite and Calcite by Sodium Silicate

**DOI:** 10.3390/ma15134699

**Published:** 2022-07-05

**Authors:** Zhenhao Guan, Kuanwei Lu, Ying Zhang, Hu Yang, Xiaokang Li

**Affiliations:** 1 Faculty of Land Resource Engineering, Kunming University of Science and Technology, Kunming 650093, China; gsc98521@163.com (Z.G.); yanghukmust@163.com (H.Y.); jealousy0722@163.com (X.L.); 2 Meishan Shunying Power Battery Materials Co., Ltd., Meishan 620000, China; lkwkmust@163.com; 3 State Key Laboratory of Complex Nonferrous Metal Resources Clean Utilization, Kunming 650093, China; 4 Yunnan Key Laboratory of Green Separation and Enrichment of Strategic Mineral Resources, Kunming 650093, China

**Keywords:** manganese ion, addition sequence, scheelite, calcite, sodium silicate, sodium oleate

## Abstract

The flotation separation (FS) of both scheelite and calcite minerals with similar physicochemical properties remains challenging, since the Ca active sites exist on their surfaces. The present work investigated the effects of different addition points of MnCl_2_ on the FS of scheelite and calcite by micro-flotation tests, zeta potential measurements, UV-Vis spectrophotometer measurements, infrared spectrum analysis, and X-ray photoelectron spectroscopy (XPS) tests, and the mechanism of separation is elucidated. Interestingly, the recovery of scheelite was 91.33% and that of calcite was 8.49% when MnCl_2_ was added after sodium silicate. Compared with the addition of MnCl_2_ before Na_2_SiO_3_, the recovery of scheelite was 64.94% and that of calcite was 6.64%. The sequence of adding MnCl_2_ followed by Na_2_SiO_3_ leads to the non-selective adsorption of Mn^2+^ on the surface of scheelite and calcite firstly, and later, sodium silicate will interact with it to produce hydrophilic silicate. This substantially enhances the hydrophilicity on the surface of both minerals, making separation impossible. In contrast, the addition of MnCl_2_ after sodium silicate can promote the formation of a metal silicate and enhance the selectivity and inhibition effect on calcite. Meanwhile, under this dosing sequence, the adsorption of Mn^2+^ on the scheelite surface offered more active sites for sodium oleate, which improved the scheelite surface hydrophobicity. This leads to a great improvement of the FS effect of scheelite and calcite.

## 1. Introduction

Tungsten(W) has outstanding properties and is an important metal resource in military, medical, mechanical, and chemical fields [1,2,3]. One of the main sources of W is scheelite (CaWO_4_), which is often related to some calcium-bearing vein minerals, mainly fluorite (CaF_2_), apatite (Ca_10_(PO_4_)_6_F_2_), and calcite (CaCO_3_) [4,5]. Currently, scheelite is mainly recovered and utilized by froth flotation [6,7]. The similar crystal structure of scheelite and its vein minerals and the similar surface chemistry of calcium atoms on the solvation [8,9,10,11] make the separation of calcium-bearing minerals a persistent problem.

In flotation processes, flotation separation (FS) between minerals is usually achieved by adding different flotation reagents that change the wettability of mineral surfaces and thus separate the minerals [9,12]. The flotation process of scheelite uses inhibitors such as sodium silicate (water glass), modified water glass, carboxymethyl cellulose, phosphoric acid, and phosphate [7,13,14,15]. The hydrophilicity of the vein mineral surface is enhanced by the interaction of functional groups in the inhibitors with Ca^2+^ on their surfaces through adsorption or generation of new hydrophilic sites [8], achieving the inhibition effect. Meanwhile, the scheelite flotation is trapped using fatty acid traps, including NaOL, oxidized paraffin soap, etc. [14,15]. The ions in these fatty acid traps react with Ca^2+^ on the mineral surface to form (Ca(OL)_2_), enhancing the floatability of the mineral [16]. In addition, isohydroxamic acid and its metal ion complexes [11,17,18], which have high trapping properties, are also gradually applied to scheelite flotation.

Metal ions still play an important role in flotation, as they can achieve enhanced inhibition or activation of the target mineral. Moreover, these metal ions, when used in combination with chemicals, enhance the selectivity of the chemicals for minerals [19]. By pre-interacting Pb(II) with scheelite, Dong et al., found that it could increase the active site of NaOL adsorption on the scheelite surface, and, hence, the coverage of NaOL, in turn [4]. Meanwhile, when Pb-water glass and Al-Na_2_SiO_3_ are used as combined inhibitors, the metal silicate polymers adsorb more on the calcite surface and less on the scheelite surface [5,20]. The flotation rate of scheelite can also be improved by using a combination of Pb-BHA and Fe-BHA collectors [11,21,22]; however, their widespread application is hindered due to the high cost. The meticulous literature study reveals that most of the previous studies have centered around the effect of metal ions on enhancing the selectivity and catchability of the agents, while ignoring that different orders of agent action on the mineral surface also bring different flotation effects. The results show that adding some metal ions promotes the chemical reaction between metal ions and silicate substances, the generation of silicate substances Si(OH)_4_, and the generation of more selective and inhibitory metal silicate substances, enhancing the inhibition of calcite [23,24]. In specific, no in-depth study on the effect of the order of adding agents in the flotation process was reported.

Therefore, in this work, the effect of different MnCl_2_ addition points on the flotation recovery (FR) of scheelite and calcite was studied. The effects of different addition points of Mn^2+^ on the flotation behavior of scheelite and calcite were studied by single mineral flotation tests. The interaction mechanism of Mn^2+^ with the surface of scheelite and calcite under the conditions of different addition points of Mn^2+^ was further investigated by a zeta potential test, FT-IR, adsorption amount determination, and XPS analysis.

## 2. Experimental Section

### 2.1. Materials

**Figure 1:**
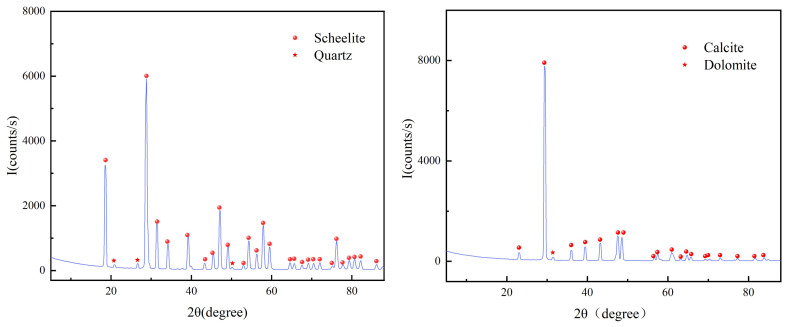
XRD patterns of pure minerals.

**Figure 2:**
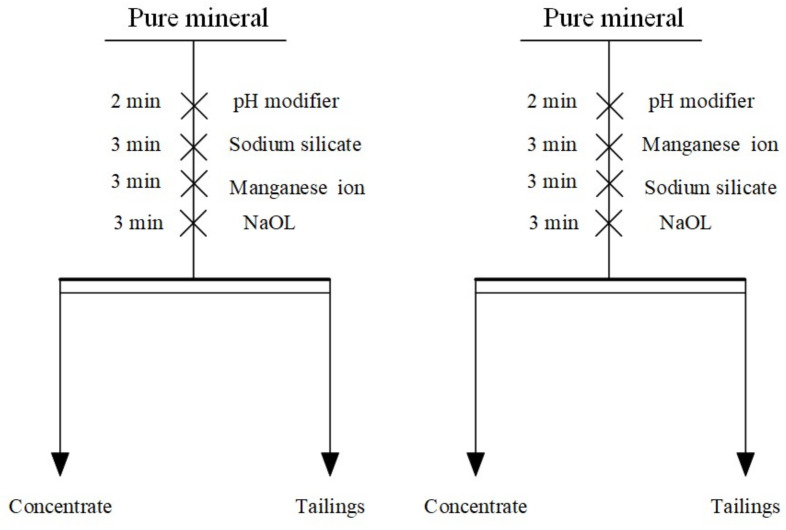
Flow chart for pure mineral flotation.

The pure mineral scheelite was taken from Qinghai, China, and the pure mineral calcite was taken from Changsha, China. Ore samples of −74 + 38 μm were collected for flotation tests. The −38 μm particle size samples were further ground to −5 μm for X-ray diffraction, IR spectroscopy, and XPS analysis. The XRD analysis of scheelite and calcite is indicated in Figure 1 and Figure 2.

The XRD analysis results of scheelite and calcite mineral samples show that both samples are high purity with no impurity peaks, and both meet the requirements of the pure mineral flotation test.

### 2.2. Micro-Flotation

The flotation tests were performed in a 40 mL FGC-type hanging tank flotation machine at an impeller speed of 1630 r/min. First, 2.0 g of the mineral sample was weighed into the 40 mL flotation tank, 36 mL of deionized water was added, and the pulp was stirred for 1 min to ensure it was as uniform as possible. Then, the corresponding flotation chemicals were added, as indicated in Figure 2. The flotation scraping time was 3 min. The froth product scraped out is the concentrate, and the remaining product in the tank is the tailings. The concentrate and tailings were filtered and weighed after being dried.

### 2.3. Zeta-Potential Measurement

Zeta potential assays were carried out by a potential analyzer (Zen-3700, Malvern Instrument Ltd., United Kingdom). A 20 mg sample was weighed in a beaker each time. Then an electrolyte sodium chloride solution of 40 mL (5 × 10^−3^ mol/L) was added and stirred with a magnetic stirrer (800 r/min) for 2 min, then the agent was added according to the agent regime of flotation test. It was stirred for 15 min and left for 5 min; the supernatant was pipetted out and put into the electrophoresis chamber for potential measurement, keeping the same conditions. Each sample was tested 3 times, and the average value was calculated as the experiment result.

### 2.4. UV–Vis Measurement

The assay was performed with an ultraviolet-visible spectrophotometer (UV-2700). First, standard solutions of different concentration gradients of NaOL were configured separately, and deionized water was used as a comparison for scanning. The absorbance of different concentrations of NaOL was measured at the stable UV absorption peak (λ = 186.8 nm), and the relationship between NaOL and absorbance was plotted by making a dotted line.

Then, 2 g of the mineral sample and 40 mL of deionized water were mixed, the agents were added in turn, stirred and left to stand for the same time. The supernatant for absorbance determination was taken, and the adsorption amount can be calculated by the formula:

(1) $$\tau =\frac{\left(C_{0}-C\right)V}{1000\;m}$$

where: *τ* is the agent’s adsorbed amount (mol/g); *C*_0_ is the initial agent concentration (mol/L); *C* is the agent concentration after action (mol/L); *V* is the solution volume (mL); *m* is the sample mass (g).

### 2.5. FTIR Tests

A Nicolet FTIR-740 FTIR spectrometer was adopted. The −2 μm mineral sample was taken 2.0 g at a time, ultrasonically cleaned for 1 min, and followed by a replenishment of deionized water to 40 mL, with the addition of chemicals according to the flotation chemicals regime and full stirring for 30 min. It was then left for 20 min, then rinsed three times with deionized water of the same pH, filtered, and dried. Grinding and mixing of KBr powder and samples, pressed using a tablet press, occurred and was subsequently tested.

### 2.6. XPS Tests

XPS testing was performed using a multifunctional scanning imaging photoelectron spectrometer model PHI5000 Versaprobe-II (Al target Kα rays as the X-ray emission source). The pulp pH was adjusted, and flotation chemicals were added sequentially in accordance with the flotation test situation, followed by rinsing the mineral samples 3–5 times with deionized water, filtering, and drying. MultiPak software was used to process and analyze the experimental data.

### 2.7. Calculations of Solution Chemistry

The hydrolysis composition distribution of Mn^2+^ and Na_2_SiO_3_ at room temperature (298.15 K) was calculated by Make Equilibrium Using Sophisticated Algorithms (MEDUSA). Analysis of the reaction process and action mechanism of flotation agents in the flotation process based on flotation solution chemistry.

## 3. Results and Discussion

### 3.1. Micro-Flotation Study

**Figure 3:**
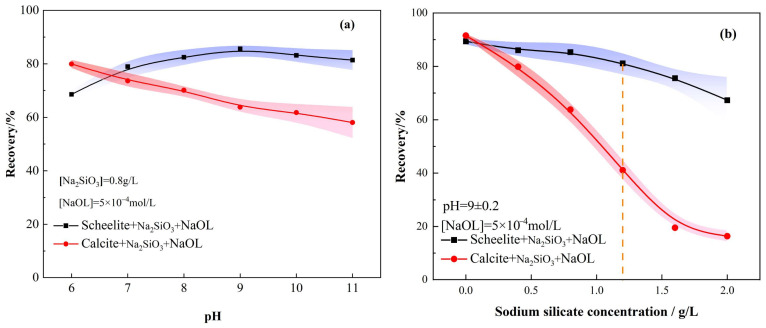
Effect of Na_2_SiO_3_ on the flotation recoveries of minerals as a function of the solution pH value (**a**) and Na_2_SiO_3_ dosage (**b**).

The effect of pH on the recovery rates is presented in Figure 3a. The recovery of scheelite increased and then decreased as pH increases with the addition of sodium silicate as an inhibitor, while calcite showed a continuous decreasing trend and stabilized. The difference between scheelite and calcite recoveries was the largest at pH = 9 ± 0.2, and, thus, was subsequently selected as the optimal pH to study the effect of Na_2_SiO_3_ dosage on the recoveries of both minerals. The effect of Na_2_SiO_3_ dosage on the recovery rates is indicated in Figure 3b. The scheelite recovery decreased gradually with the increase of Na_2_SiO_3_ dosage; it shows that when too much Na_2_SiO_3_ is used, it will also adsorb with scheelite, thus reducing the FR of scheelite [25]. Relatively speaking, the inhibition effect of Na_2_SiO_3_ on calcite was more obvious. Among all the Na_2_SiO_3_ dosage contents, 1.2 g/L showed a better separation effect (As shown by the dotted line in the Figure 3b). The FR of the two minerals differed by 40.03%; however, it is insufficient to effectively separate scheelite and calcite. Therefore, the separation effect of both minerals was enhanced by adding metal ions.

**Figure 4:**
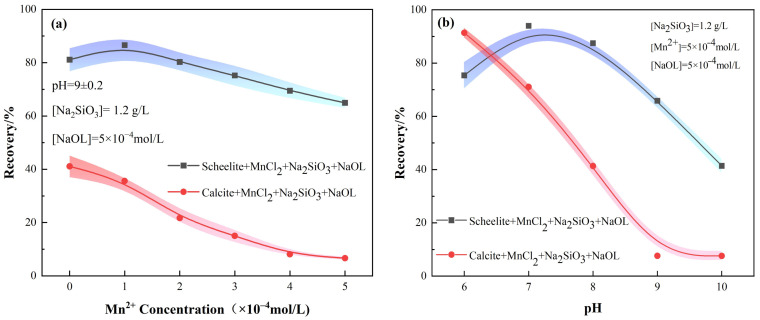
Effect of MnCl_2_ on mineral FR under the addition sequence of MnCl_2_^+^ Na_2_SiO_3_. (**a**) MnCl_2_ dosage; (**b**) pH.

**Figure 5:**
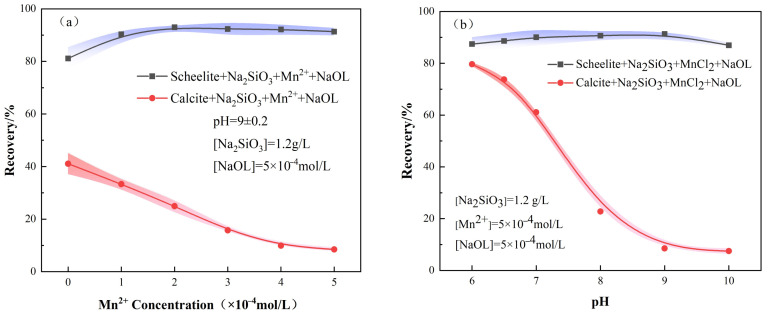
Effect of MnCl_2_ on mineral FR under the addition sequence of Na_2_SiO_3_^+^ MnCl_2_. (**a**) MnCl_2_ dosage; (**b**) pH.

The effect of adding MnCl_2_ at different stages on the flotation recoveries of the two minerals is indicated in Figure 4 and Figure 5, and Figure 4b represents the recovery rates when MnCl_2_ was added first, followed by Na_2_SiO_3_. The scheelite recovery first increased and then decreased significantly with the increase of pH, while the recovery of calcite gradually decreased and then flattened out. It can be seen that the best separation efficiency of the two minerals was achieved at pH = 9 ± 0.2. On the other hand, when the dosing sequence was Na_2_SiO_3_ followed by MnCl_2_, the recovery of scheelite tended to increase gradually as pH increases, reaching the maximum at pH = 9 ± 0.2, after which it started decreasing. In contrast, the recovery of calcite gradually decreased and then leveled off, similar to that in the other dosing sequence with MnCl_2_ + Na_2_SiO_3_.

Furthermore, the relationship between Mn^2+^ concentration and mineral FR was also investigated. For the target mineral scheelite, it is obvious from Figure 4a that the recovery of scheelite firstly increases with the addition sequence of MnCl_2_ + Na_2_SiO_3_, and then decreases continuously with the increase of the Mn^2+^ concentration. However, when Na_2_SiO_3_ was added first, the recovery of scheelite increased first and then leveled off at a concentration of Mn^2+^ of 5 × 10^−4^ mol/L (Figure 5a). The separation efficiency of scheelite and calcite under this condition was the best, with a difference of 82.84% in recovery.

As for the calcite, after the addition of MnCl_2_, its FR was lower than the condition of Na_2_SiO_3_ alone, regardless of the addition order, indicating that the addition of MnCl_2_ contributes to the selective inhibition of calcite by Na_2_SiO_3_. As the Mn^2+^ dosage increases, the recovery of calcite gradually decreased and then leveled off. Therefore, the dosing sequence of Na_2_SiO_3_ + MnCl_2_ can separate the two minerals more effectively.

### 3.2. Zeta Potential Analysis

**Figure 6:**
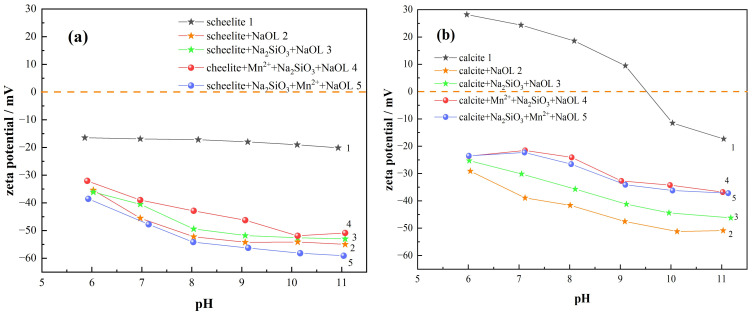
Effect of different agent regimes on the surface potential of two minerals. (**a**) Scheelite; (**b**) calcite.

This assay can be used to analyze the adsorption behavior of reagents on the surface of mineral particles during flotation and the effect on the surface charge of minerals [26]. The effect of pH on the surface zeta potential of mineral particles of scheelite and calcite is given in Figure 6.

Figure 6a indicates that the zero electric point is not observed in scheelite throughout the pH interval and the surface potentials are negative [12]. The iso-electric point (IEP) of the surface of calcite was obtained at around pH = 9.5 (Figure 6b), which was consistent with previous literature studies [13,27].

Obviously, when NaOL interacts with minerals, the potentials all tend to show a negative shift (line 2 in Figure 6), indicating that the interaction of the oleate ion (RCOO^−^) with the mineral surface in the anion collector NaOL leads to a transfer in the negative direction, as evidenced by previous studies by some scholars [26,28]. In contrast, when Na_2_SiO_3_ was added (line 3 in Figure 6), The mineral particle surface potentials of both scheelite and calcite are shifted in the positive direction; however, the shift of the scheelite potential is smaller. The results of the previous flotation tests also confirm this, indicating that sodium silicate would interact more with the calcite surface; thus, it prevents NaOL from reacting with its surface and inhibits its floating [27].

When Mn^2+^ was added first (line 4 in Figure 6a), The scheelite potential was positively shifted by 5.5 mV at pH = 9 compared with the case of adding only Na_2_SiO_3_. The increase of sodium silicate adsorption may decrease the adsorption of NaOL on the mineral surface. On the other side, when the MnCl_2_ is added later (line 5 in Figure 6a), the scheelite potential is clearly the most negatively shifted compared to all other dosing conditions and shifted in the negative direction by 4.5 mV when only sodium silicate was added. Referring to the flotation test results, which showed an increase in recovery compared with the addition of Na_2_SiO_3_ only, it is obvious that the post-addition of Mn^2+^ promotes the activity of the scheelite surface and causes more oleate ions to react with the surface of scheelite by adsorption, thus contributing to the negative shift trend of surface potential.

After adding Mn^2+^ first (line 4 in Figure 6b), the calcite surface potential is positively shifted by 8.5 mV at pH = 9 when Mn^2+^ was added first, more than that when only sodium silicate was added, due to the lower adsorption of oleate ions. The calcite surface potential was slightly shifted in the negative direction when Mn^2+^ was added later (line 5 in Figure 6b), more than when Mn^2+^ was added first. The recovery was slightly higher when Mn^2+^ was added first. The addition of Mn^2+^ first provides active sites for sodium silicate, resulting in a slight enhancement of the calcite surface hydrophilicity. Therefore, the dosing sequence of adding Na_2_SiO_3_ first and then Mn^2+^ was chosen as the best condition.

### 3.3. UV–Vis Analysis

**Figure 7:**
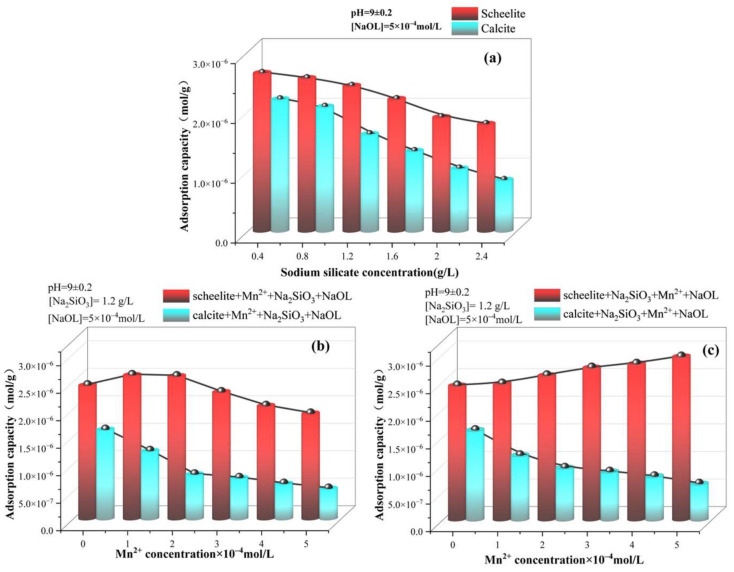
Effects of different agent concentrations and dosing sequences on the adsorption of sodium oleate on the surface of two minerals. (**a**) Na_2_SiO_3_; (**b**) MnCl_2_ + Na_2_SiO_3_; (**c**) Na_2_SiO_3_ + MnCl_2_.

The effect of adding different agents and varying dosages is presented in Figure 7a. The addition of sodium silicate resulted in an inhibition effect in both minerals; however, the inhibition effect on calcite was more obvious. The adsorption capacity of NaOL on both mineral surfaces showed a negative correlation with the dosage of Na_2_SiO_3_. The increase of sodium silicate dosage makes it impossible to achieve FS of two minerals.

The effect of increasing the Mn^2+^ concentration on the adsorption amount of NaOL when MnCl_2_ was added first is shown in Figure 7b. An increasing trend was observed with the increasing dosage of Mn^2+^ when MnCl_2_ was added first, compared to that without MnCl_2_ addition. This may be due to the fact that at low Mn^2+^ concentrations, NaOL will preferentially adsorb on the scheelite surface before Na_2_SiO_3_ due to the poor adsorption of sodium silicate itself to scheelite, causing an increase in adsorption. On the other hand, the increase of Mn^2+^ dosage led to more active sites of Mn^2+^ adsorption on the scheelite surface, and, thus, the adsorption of Na_2_SiO_3_ also increased, which increased the hydrophilicity of the surface. In addition, the adsorption of NaOL on scheelite showed a decreasing trend, indicating that the increase of Mn^2+^ dosage would inhibit the flotation of scheelite. In addition, the adsorption of NaOL on calcite also showed a decreasing trend, leading to the inability of the two minerals to be separated.

As shown in Figure 7c, the addition of MnCl_2_ after Na_2_SiO_3_ increased the adsorption of C_18_H_33_NaO_2_ on the scheelite surface gradually as the Mn^2+^ concentration increases. The NaOL content of the calcite surface gradually decreased and did not vary much under the two dosing orders, which was consistent with the results of the flotation test and zeta potential. At the dosage of Mn^2+^ of 5 × 10^−4^ mol/L, the difference in the C_18_H_33_NaO_2_ adsorption amount between the two minerals reached 2.306 × 10^–6^ mol/g. It indicates that the dosing sequence of adding Mn^2+^ after sodium silicate can facilitate the FS of the two minerals.

### 3.4. FTIR Analysis

The IR spectral method is used to study the nature and phenomena of adsorption of substances at the solid–liquid interface to study the nature of adsorption of flotation chemicals on mineral surfaces during flotation, as well as the orientation of adsorbed substances and detailed information on adsorption/resolution equilibria and substance formation [29]. The mechanism of the action of the agents on the surface of the two minerals under the conditions of two incorporation points of manganese ions was analyzed by infrared spectroscopy.

**Figure 8:**
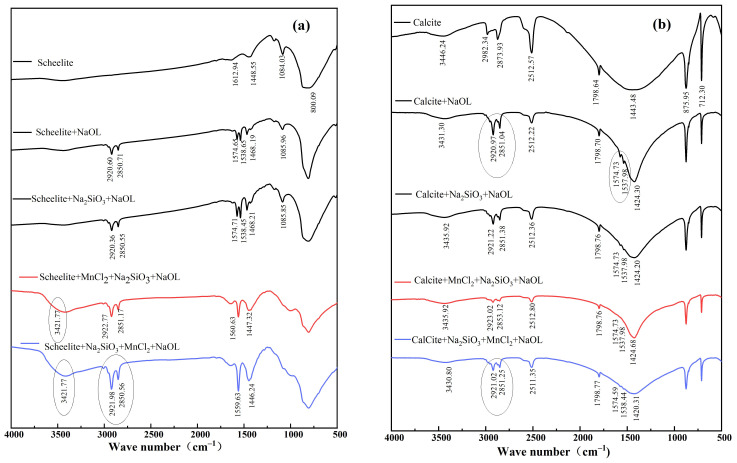
Infrared spectra of mineral surfaces before and after the action of different agents. (**a**) Scheelite; (**b**) calcite.

The IR spectra before and after the addition of agents are displayed in Figure 8. In the presence of added sodium oleate, new absorption peaks were observed at 2920.60 cm^−1^ and 2850.71 cm^−1^, indicating the adsorption of C_18_H_33_NaO_2_ onto the scheelite surface, respectively, and the absorption peaks at 1574.65 cm^−1^, 1538.65 cm^−1^, and 1468.19 cm^−1^ due to the symmetric and asymmetric stretching vibrations of carboxyl [30]. It indicates that NaOL is adsorbed on the scheelite surface. The absorption peaks at 2920.36 cm^−1^ and 2850.55 cm^−1^ were attributed to -CH_2_-symmetric stretching peaks after the interaction of Na_2_SiO_3_ and NaOL with minerals [31]. After adding Mn^2+^ in a different order, a new peak appeared in the IR spectrum at 3421.77 cm^−1^ on the scheelite surface, due to the interaction of MnCl_2_ with the surface of the scheelite mineral. When Mn^2+^ was added after sodium silicate, the absorption peaks at 2921.98 cm^−1^ and 2850.56 cm^−1^, because of the antisymmetric stretching peak of alkyl CH_2_, and the absorption peaks at 1559.63 cm^−1^ and 1446.24 cm^−1^, are attributed to the -COO absorption vibration peak. Meanwhile, the absorption peaks of alkyl CH_2_ and COO- appearing on the scheelite surface are significantly enhanced in intensity compared with those under other agent regimes. The results showed that Mn^2+^ added after sodium silicate could adsorb onto the scheelite surface and enhance its Mn^2+^ active site; thus, the adsorption capacity of NaOL on the scheelite surface was enhanced.

After adding NaOL (Figure 8b), the absorption peaks at 2920.97 cm^−1^ and 2851.04 cm^−1^ because of the antisymmetric stretching peak of CH_2_. The absorption peak of calcite at 1443.48 cm^−1^ is shifted to 1424.30 cm^−1^. After the addition of Mn^2+^ after sodium silicate, the absorption peaks on the calcite surface at 2921.02 cm^−1^ and 2851.25 cm^−1^, because of the antisymmetric stretching peaks of alkyl CH_2_, and the peaks at 1572.53 cm^−1^ and 1534.69 cm^−1^ were attributed to the COO-absorption vibration peaks. In comparison with the IR spectra of calcite after the action of the other three agent regimes, the absorption peaks of alkyl CH_2_ and COO- were also present on the calcite surface, but their intensities were greatly weakened. Combined with the results of the adsorption amount test, and obviously, after the addition of Na_2_SiO_3_, they preferentially adsorb with calcite, and then after the addition of manganese chloride, there is more free Mn^2+^ in the solution due to the physical and chemical adsorption of calcite surface with sodium silicate, followed by the inability to adsorb Mn^2+^. After adding sodium oleate, more oleate is generated by the reaction between Mn^2+^ and sodium oleate, which makes the calcite-weak sodium oleate absorption peaks appear on the surface.

The IR spectra of the two mineral surfaces were analyzed together, and the two dosing orders of MnCl_2_ had different effects on the adsorption of inhibitors and traps on the surfaces of the two minerals. The dosing sequence of adding MnCl_2_ after Na_2_SiO_3_ can enhance the adsorption capacity of NaOL to scheelite and reduce the adsorption capacity of NaOL to calcite, thus enabling the two minerals to be separated.

### 3.5. XPS Analysis

**Figure 9:**
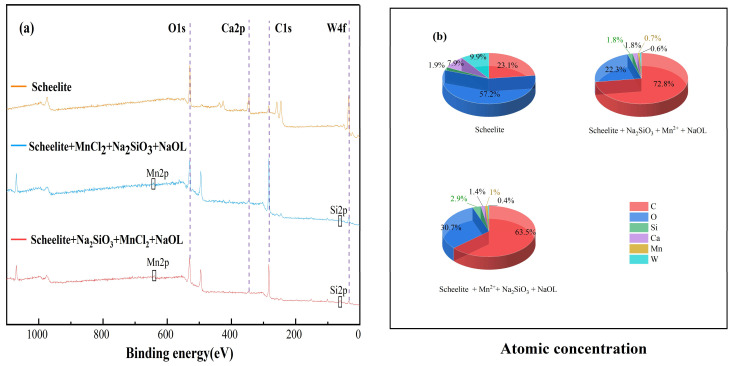
XPS survey scan and atomic concentrations of scheelite under different dosing sequences. (**a**) XPS total spectrum; (**b**) atomic concentration.

The XPS spectra of scheelite under different dosing sequences (Figure 9a) show that the characteristic peaks of Mn and Si appear under two different dosing sequences (I, MnCl_2_ + Na_2_SiO_3_ + NaOL; II, Na_2_SiO_3_ + MnCl_2_ + NaOL), which led to the conclusion that Mn and Si would adsorb to the surface of scheelite under these two conditions. However, the peak intensity of C1s in II was obviously larger than that of I. The atomic concentration statistics (Figure 9b) of C in the II order increased by 9.3% compared to those of I. Moreover, the atomic concentration of Si under the II condition is 1.8%, which is 1.1% lower than that of I, indicating that the I order can lead to more sodium silicate acting on the scheelite surface, leading to the enhancement of hydrophilicity on the scheelite surface. This may be because in condition I (when Mn^2+^ was added first), Mn^2+^ with more active sites adsorbs on the surface, and subsequently, after adding sodium silicate, reacts with silicate to generate more metal silicates adsorbed on the scheelite surface. In condition II (when sodium silicate was added first), sodium silicate is less selective, and thus less adsorbed on scheelite. After adding MnCl_2_, Mn^2+^ acts on the surface of minerals, and when sodium oleate is added, it can better act with scheelite to strengthen the flotation effect of scheelite.

**Figure 10:**
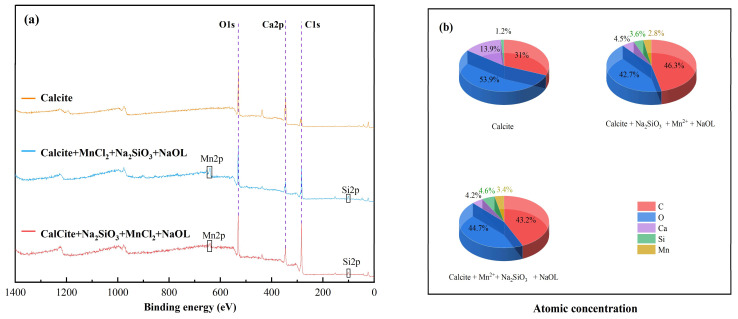
XPS survey scan and atomic concentrations of calcite under different dosing sequences. (**a**) XPS total spectrum; (**b**) atomic concentration.

The XPS spectra of calcite under different dosing sequences (Figure 10a) show that the characteristic peaks of Mn and Si were generated under both conditions. Comparing the intensity of the peaks under the two dosing orders, the intensity of the peaks of C1s under the I order is lower than that in II. The atomic concentration statistics (Figure 10b) reveal that C atomic concentration in the I order is 3.1% lower than that of II, while the concentration of Si atoms is 1% more than that of II. It can be seen that MnCl_2_ acts with calcite first before Na_2_SiO_3_, which can reduce the floatability of minerals. However, when MnCl_2_ was added first, resulting in the absence of selectivity of sodium silicate for scheelite and calcite, the recovery of calcite was lower under the I sequence. It is not possible to separate scheelite and calcite by this dosing sequence. Therefore, the separation of the two minerals under the dosing sequence of Minerals + Na_2_SiO_3_ + MnCl_2_ + NaOL was better than that of sodium silicate alone.

**Figure 11:**
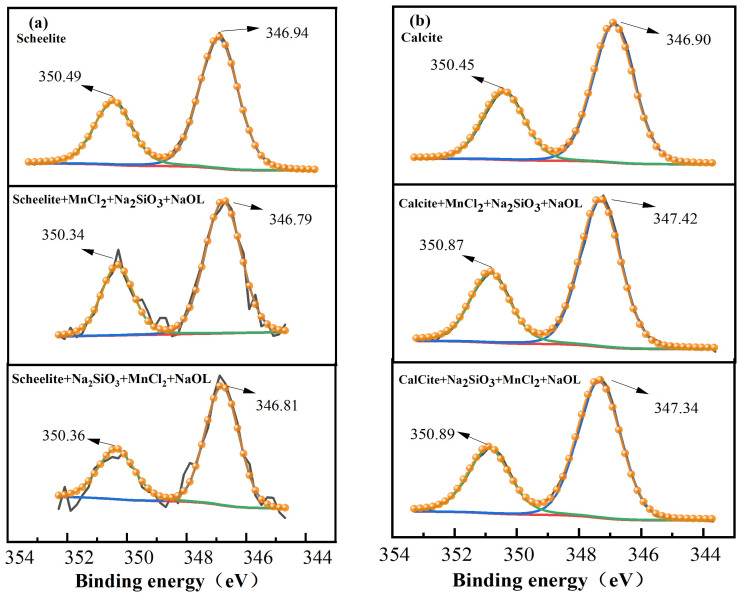
High-resolution XPS spectra of Ca2p for two minerals under different dosing sequences. (**a**) Scheelite; (**b**) calcite.

The high-resolution XPS spectra of the Ca2p of scheelite and calcite interacting with pharmaceuticals under different dosing sequences were analyzed by peak fitting and separation. It can be observed from Figure 11a that the peaks appearing at the binding energies of 346.94 eV and 350.49 eV can be assigned to Ca2p3/2 and Ca2p1/2, respectively [31]. The binding energies (BE) of Ca2p3/2 and Ca2p1/2 on the surface of scheelite after the action of MnCl_2_ + Na_2_SiO_3_ + NaOL were both shifted by 0.15 eV and after, the actions of Na_2_SiO_3_ + MnCl_2_ + NaOL were both shifted by 0.13 eV. The Ca2p BE on the surface of the scheelite had some offset, but the offset value was smaller than the error value, which indicated that the effect of chemicals on the Ca2p orbitals on the surface of the scheelite was small under the two dosing sequences.

The calcite also presented a similar spectrum for Ca2p. As can be observed from Figure 11b, the peaks Ca2p3/2 and Ca2p1/2 appeared at the binding energies of 346.90 eV and 350.45 eV, respectively [5]. The BE shift of Ca2p3/2 on the calcite surface after the action of MnCl_2_ + Na_2_SiO_3_ + NaOL was 0.52 eV, and that of Ca2p1/2 was 0.42 eV. Similarly, the BE shift of Ca2p3/2 on the calcite surface after the action of Na_2_SiO_3_ + MnCl_2_ + NaOL was 0.44 eV, and there was one for Ca2p1/2. The shift after the action of MnCl_2_ + Na_2_SiO_3_ + NaOL was more significant, indicating that the chemisorption was stronger under this dosing sequence, the results of previous flotation tests also confirm this. The Ca2p BE on the calcite surface also shifted after the action with the agent, and the shifted values were larger than the instrumental error values, indicating that the agent can react with the calcite surface.

### 3.6. Solution Chemistry Calculation Analysis

**Figure 12:**
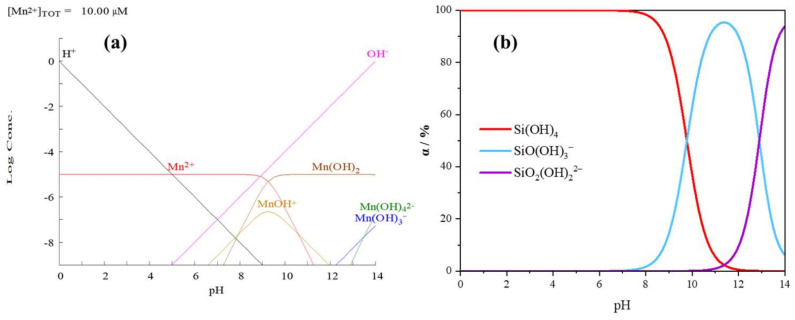
Effect of the solution’s pH value on the species distribution diagram of (**a**) monomer Mn^2+^ (C = 5 × 10^−4^ mol/L) and (**b**) silicate anions.

The effect of pH on the species formation and content of Mn^2+^, silicates in the Mn^2+^, and the silicate solution system is indicated in Figure 12. Based on Figure 12, the main chemical equations for the chemical equilibrium of Mn^2+^ and the silicate in a solution can be deduced as follows:

(2) $${\mathrm{MnCl}}_{2}⇌{\mathrm{Mn}}^{2+}+2{\mathrm{Cl}}^{-},$$

(3) $${\mathrm{Mn}}^{2+}+{\mathrm{OH}}^{-}⇌{\mathrm{MnOH}}^{+},$$

(4) $${\mathrm{Mn}}^{2+}+2{\mathrm{OH}}^{-}⇌{\mathrm{MnOH}}_{2}\;,$$

(5) $${\mathrm{Mn}}^{2+}+3{\mathrm{OH}}^{-}⇌\mathrm{Mn}{\left(\mathrm{OH}\right)}_{3}^{-},$$

(6) $${\mathrm{Mn}}^{2+}+4{\mathrm{OH}}^{-}⇌\mathrm{Mn}{\left(\mathrm{OH}\right)}_{4}{}^{2-},$$

(7) $$S_{i}O_{2}+2H_{2}O⇌S_{i}{\left(0H\right)}_{4},$$

(8) $$S_{i}{\left(\mathrm{OH}\right)}_{4}⇌S_{i}O{\left(\mathrm{OH}\right)}_{3}^{-}+H^{+},$$

(9) $$S_{i}O{\left(\mathrm{OH}\right)}_{3}^{-}⇌S_{i}O_{2}{\left(\mathrm{OH}\right)}_{2}^{2-}+H^{+}.$$

Figure 12a shows the gradual hydrolysis of Mn^2+^ in the solution and the generation of four manganese-containing components: MnOH^+^, $\mathrm{Mn}{\left(\mathrm{OH}\right)}_{2}$, $\mathrm{Mn}{\left(\mathrm{OH}\right)}_{3}^{-}$, and $\mathrm{Mn}{\left(\mathrm{OH}\right)}_{4}{}^{2-}$. When the pH < 9.29, Mn^2+^ dominates the composition. After increasing the pH, $\mathrm{Mn}{\left(\mathrm{OH}\right)}_{2}$, $\mathrm{Mn}{\left(\mathrm{OH}\right)}_{4}{}^{2-}$, and $\mathrm{Mn}{\left(\mathrm{OH}\right)}_{3}^{-}$ dominate the system. Figure 12b shows that there are three main components in the Na_2_SiO_3_ solution. Si(OH)_4_ dominates when the pH < 9.6, and $S_{i}O{\left(\mathrm{OH}\right)}_{3}^{-}$ and $S_{i}O_{2}{\left(\mathrm{OH}\right)}_{2}^{2-}$ dominate when the pH < 12.8. When MnCl_2_ is added after Na_2_SiO_3_, the hydrolysis of Mn^2+^ consumes lots of OH^−^. Consequently, the concentration of H^+^ in the solution increases and Equations (8) and (9) move in the opposite direction, leading to the production of more Si(OH)_4_. Si(OH)_4_ is the active component that inhibits calcite flotation since it selectively acts on calcite and interferes with the adsorption of NaOL on its surface, thus reducing the recovery during calcite flotation [23,27]. When MnCl_2_ is added before Na_2_SiO_3_, Mn^2+^ is pre-sorbed on the surface of both minerals, and when Na_2_SiO_3_ is subsequently added, the silicate hydrolysis products interact with the mineral surface through physical adsorption, resulting in both scheelite and calcite being inhibited from floating.

**Figure 13:**
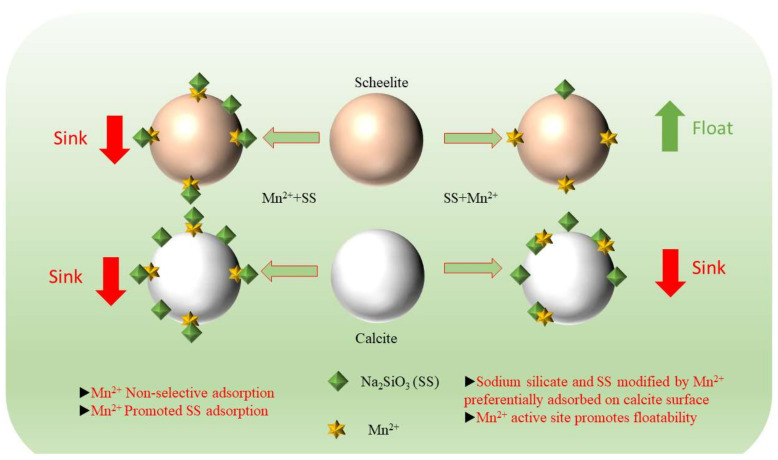
Mechanism of interaction on the surface of scheelite and calcite under different addition sequences of MnCl_2_.

The mechanism of the interaction between two different addition points of MnCl_2_ and mineral surfaces is shown in Figure 13. Under the additional order of MnCl_2_ + Na_2_SiO_3_, Mn^2+^ will be adsorbed on both mineral surfaces due to its lack of selectivity, increasing the active sites on both minerals surfaces. This resulted in enhanced adsorption of sodium silicate on both minerals surfaces., which led to the enhancement of hydrophilicity on both minerals surfaces. This inhibited the minerals, and separation could not be achieved. In contrast, under the dosing sequence of Na_2_SiO_3_^+^ MnCl_2_, sodium silicate was added first, and Na_2_SiO_3_ selectively adsorbed on calcite surface, while it adsorbed less on scheelite. With the subsequential addition of MnCl_2_, Mn^2+^ reacted with the surface of scheelite and adsorbed on its surface, thus increasing the active site of NaOL on its surface. It also reacted with free sodium silicate in the pulp to form a more selective metal silicate; as a result, the inhibition effect on calcite was enhanced.

## 4. Conclusions

In this work, the separation efficiency of scheelite and calcite was studied under different dosing orders of MnCl_2_ and Na_2_SiO_3_. Through micro-flotation tests, it can be seen that different addition points of MnCl_2_ lead to different flotation results (I, MnCl_2_ + Na_2_SiO_3_ + NaOL; II, Na_2_SiO_3_ + MnCl_2_ + NaOL). The recovery of scheelite and calcite was lower in condition I. Furthermore, the suppression efficiency of the agent on calcite was clearly better than that on scheelite under the II condition, and a small activation effect on scheelite was also observed. The results of the zeta potential analysis show that the interaction of NaOL and the mineral surface results in a negative shift of the potential, and condition I led to a positive shift in the surface potential of scheelite compared to the effect of Na_2_SiO_3_ alone. On the other hand, in the II condition, the shift was in the negative direction. Combined with the analysis of UV-Vis results, under condition I, the effect of NaOL interaction with both scheelite and calcite showed a decreasing trend, while the adsorption of C_18_H_33_NaO_2_ was enhanced on scheelite and weakened on calcite under condition II. FTIR and XPS analyses show the adsorption of Mn on the surface of both minerals under the two different addition point conditions of MnCl_2_. By comparing the Si atom concentration, condition I was significantly higher than condition II, indicating that Na_2_SiO_3_ has a stronger effect with scheelite in condition I. This inhibits the flotation of scheelite and makes the separation process difficult. In contrast, the increase of C1s peak on the surface of scheelite and the increase of the C atom concentration under condition II indicates that the adsorption of NaOL on the scheelite surface was higher. The adsorption of Na_2_SiO_3_ on both minerals surfaces is promoted under condition I, leading to the inhibition of both minerals. In contrast, under condition II, the hydrophilic silicate and the generated metal silicate material dissolved in the slurry selectively adsorb on the calcite surface, increasing the calcite surface hydrophilicity. More Mn^2+^ and less Na_2_SiO_3_ adsorb with scheelite, providing Mn active sites for the adsorption of NaOL. Conclusively, the dosage sequence plays a significant role in the separation process of scheelite and calcite and provides a new research idea and effective method for the separation of scheelite and calcite.
